# Supplementary material for: Period-doubling in the phase dynamics of a shunted HgTe quantum well Josephson junction
Source: Nat Commun. 2025 Mar 29;16:3068. doi: 10.1038/s41467-025-58299-z (PMC11954865; doi:10.1038/s41467-025-58299-z)
Supplement: Supplementary file 1 — Supplementary Information [file 41467_2025_58299_MOESM1_ESM.pdf]

Supplementary Information: Period-doubling in the phase dynamics of a shunted HgTe quantum well Josephson junction

Wei Liu,[1,2] Stanislau U. Piatrusha,[1,2] Xianhu Liang,[1,2] Sandeep Upadhyay,[1,2] Lena Fürst,[1,2] Charles Gould,[1,2] Johannes Kleinlein,[1,2] Hartmut Buhmann,[1,2] Martin P. Stehno,[1,2] and Laurens W. Molenkamp[1,2]

[1]*Physikalisches Institut (EP3), Universität Würzburg, Am Hubland, 97074 Würzburg, Germany.*

[2]*Institute for Topological Insulators, Universität Würzburg, Am Hubland, 97074 Würzburg, Germany*

S1. DC CHARACTERIZATION

In this section, we provide additional information on $I-V$ hysteresis and Shapiro step measurements on the Josephson junction without external shunt resistor.

S1.1. Hysteresis in the current-voltage characteristic

The $I-V$ traces of the device without external shunt resistor [data in Fig. 1 of the main text] are hysteretic for large values of the critical current, I_c. The junction exhibits abrupt, stochastic switching into the finite voltage state at the switching current, $I_\mathrm{sw} \lessapprox I_\mathrm{c}$, and a transition back into the zero-voltage state at the retrapping current, I_r, as the bias current, I_b, is ramped from $|I_\mathrm{b}| > I_\mathrm{c}$ back to zero. The gate-voltage-dependencies of I_sw and I_r are plotted in Fig. S1. The values I_sw and I_r are extracted using a voltage criterion $|V| < 2.5\,\mu\mathrm{V}$. As we conducted DC characterization for different gate voltage ranges (V_g) in separate measurements, the data in Fig. S1 are assembled from five individual V_g-sweeps. For two datasets, we correct the gate voltage value by $-10\,\mathrm{mV}$ to account for

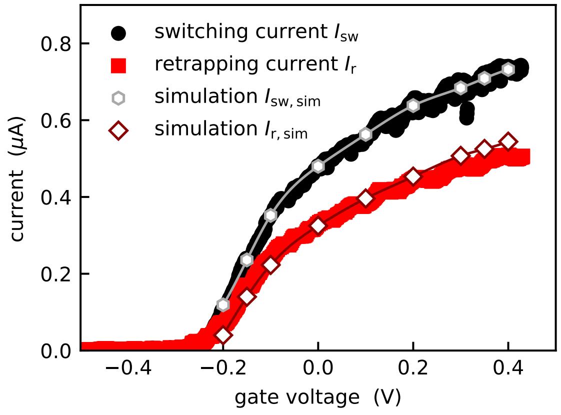

FIG. S1. **Gate voltage dependence of the I-V hysteresis -** Switching current, I_sw, and retrapping current, I_r, plotted as function of gate voltage (solid symbols). The data are fitted well by an RSCJ model simulation (open symbols) with shunt resistance $R = V_\mathrm{r}/I_\mathrm{r}$ and shunt capacitance $C = 0.28\,\mathrm{pF}$. Deviations are visible at large gate voltages when retrapping occurs for $eV_\mathrm{r} \approx hf_\mathrm{res}$, i.e., near a circuit resonance with resonance frequency f_res.

gate hysteresis that developed as we increased the gate voltage ranges. Since the switching is stochastic in nature, we plot the maximum of several I_{sw} values for a given V_{g} when V_{g}-ranges overlap. The identical procedure is used in Fig. 1e of the main text.

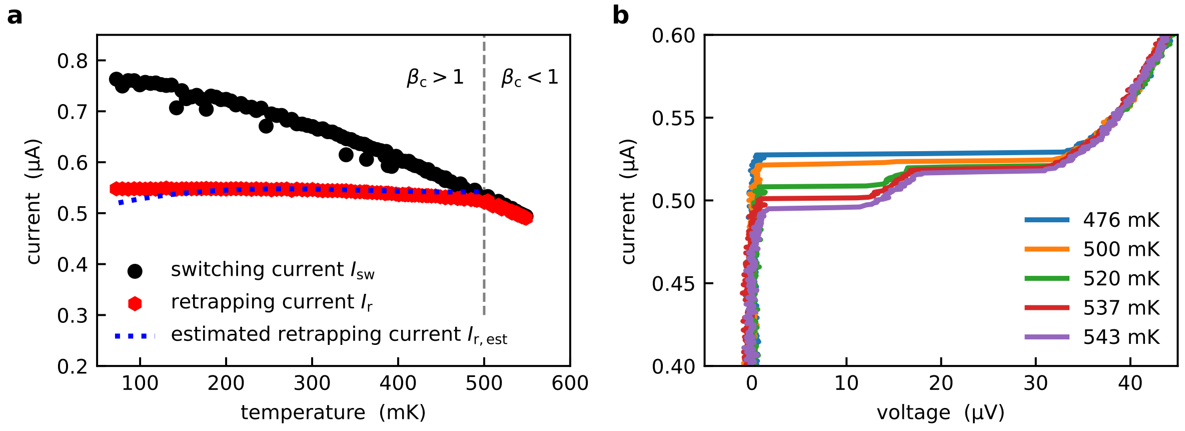

FIG. S2. **Temperature dependence of the I-V hysteresis at $V_{\mathrm{g}} = 0.5\,\mathrm{V}$ - (a)** Switching current, I_{sw}, and retrapping current, I_{r}, as a function of temperature. Hysteresis sets in when the Stewart-McCumber parameter reaches $\beta_{\mathrm{c}} \approx 1$. (**b**) Retrapping branch of the I-V characteristics for different temperatures. A second switching event occurs between the voltages $\sim 18\,\mu\mathrm{V}$ and $\sim 36\,\mu\mathrm{V}$.

The data are overlayed by the results of numerical simulations of the resistively- and capacitively-shunted junction (RCSJ) model [open symbols in Fig. S1]. For the simulations, we use the resistance values $R = V_{\mathrm{r}}/I_{\mathrm{r}}$ where V_{r} is the junction voltage recorded in the dissipative state before retrapping occurs. The value of the capacitance C is set to 0.28 pF, the identical value we use for the simulations in the main text. We further assume a finite temperature of 40 mK that is modeled by a white noise current source in parallel to the RCSJ [1]. The critical current of the junction, I_{c}, is chosen such that I_{sw} matches the experimental data. The values are $\sim 10\,\mathrm{nA}$ to $20\,\mathrm{nA}$ larger than I_{sw}. The simulations agree well with the switching behavior of the junction observed in experiment. A small deviation is visible at higher gate voltages.

To investigate further the switching behavior in this gate voltage region, we plot I_{sw} and I_{r} as a function of temperature, T, for $V_{\mathrm{g}} = 0.5\,\mathrm{V}$ in Fig. S2a. $I - V$ hystersis sets in

at $T \approx 500\,\mathrm{mK}$. Whereas I_{sw} increases upon cooling, the values of the retrapping current remain approximately constant below this temperature. We observe that the onset of $I-V$ hysteresis coincides with critical damping, i.e., the Stewart-McCumber parameter $\beta_{\mathrm{c}}(T) \approx 1$ [2, 3], as indicated by the dashed vertical line in Fig. S2a.

The retrapping current can be estimated by [4]

$$I_{\mathrm{r,est}}(T) \approx \frac{4}{\pi Q} I_{\mathrm{c}}(T)\,, \tag{S1}$$

where $Q = \sqrt{\beta_{\mathrm{c}}(T)}$ is the quality factor of the damped phase oscillations. Let us assume $I_{\mathrm{c}}(T) \approx I_{\mathrm{sw}}(T)$. We plot the result of eq. S1 as dotted blue line in Fig. S2a and find that the increase in I_{c} is compensated by a rise in Q that enters in the denominator of eq. S1, thus the retrapping current stays approximately constant below $500\,\mathrm{mK}$.

Let us remark that the dynamics of the junction are also affected by the presence of a circuit resonance in the measurement setup. It leads to features in the subgap resistance centered around the voltages $\sim 18\,\mu\mathrm{V}$ and $\sim 36\,\mu\mathrm{V}$. The interference with the junction dynamics is visible in Fig. S2b. The figure depicts the retrapping current branch of the

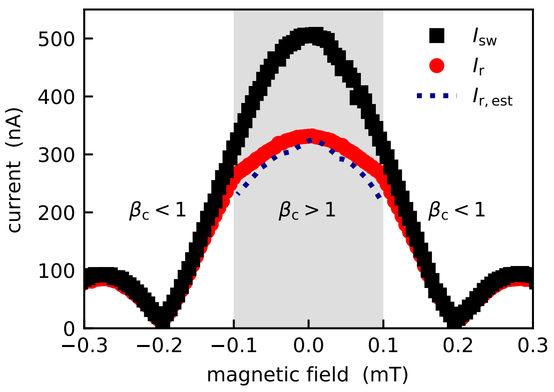

FIG. S3. **Magnetic field dependence of the $I-V$ hysteresis at $V_{\mathrm{g}} = 0$ -** Switching current, I_{sw}, and retrapping current, I_{r}, as a function of magnetic field. Hysteresis occurs in the region of underdamped junction dynamics, i.e. $\beta_{\mathrm{c}} > 1$ (grayed background). The retrapping current $I_{\mathrm{r,est}}$ is calculated based on eq. S1 assuming $I_{\mathrm{c}} \approx I_{\mathrm{sw}}$.

$I-V$ traces for different temperatures in the transition region. The traces taken at higher temperature feature two switching events, of which the second one occurs between the two characteristic voltages of the circuit resonance. Retrapping at $V = 36\,\mu\mathrm{V}$ is also visible in the Shapiro map of Fig. S4a at low microwave power. We stress that this is not a singular observation. We have tracked the presence of circuit resonances in our setup since becoming aware of this issue and found a similar interplay between junction phase dynamics and circuit resonance features in the $I-V$s of other Josephson devices. For clarity, note that we omit circuit resonances in the RSCJ simulations of Fig. S1, which accounts for the discrepancy in retrapping current at large positive gate voltages.

Finally, we explore the effect of magnetic field on the $I-V$ hysteresis for $V_{\mathrm{g}} = 0$ in Fig. S3. $I-V$ hysteresis is observed in a region with underdamped junction dynamics, i.e. $\beta_{\mathrm{c}} > 1$ [gray background]. The definitions of the parameters R, C, and β_{c} are as above. The estimated retrapping current [eq. S1, dotted blue line] agrees reasonably well with the extracted value I_{r}.

In summary, the data strongly suggest that $I-V$ hysteresis in our junction originates from the dynamics of an underdamped Josephson junction. Further, the RCSJ modeling supports our choice for the value of the geometric capacitance of the device, $C_{\mathrm{J}} = 0.28\,\mathrm{pF}$. We stress that our wiring layout is not uncommon for topological insulator- and semiconductor-based Josephson junctions. Such devices often exhibit pronounced $I-V$ hysteresis. In many cases, the effect is attributed to nonequilibrium distributions of quasiparticle states in the device ('junction heating' [5, 6]) or inhibited retrapping by quantum-mechanical tunneling of the junction phase [7] as the junction capacitance is deemed too small to cause hysteresis. Our analysis shows that the capacitance of the entire structure must be determined, including the on-chip wiring layer, before ruling out a conventional, capacitance-based mechanism of $I-V$ hysteresis.

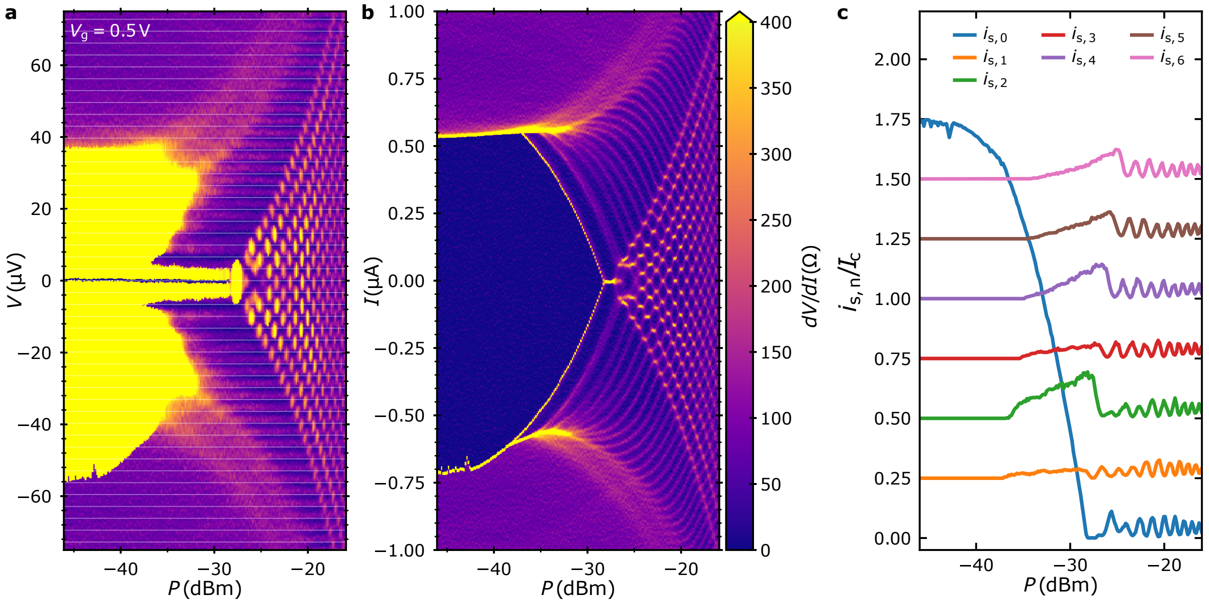

FIG. S4. **Shapiro step data for $V_g = 0.5\,\mathrm{V}$ and drive frequency $f = 1.6\,\mathrm{GHz}$ -** Maps of the differential resistance $\mathrm{d}V/\mathrm{d}I$ obtained by numerical differentiation and plotted as function of microwave power P and (**a**) voltage V or (**b**) current I. White lines mark multiples of $V_1 = hf/2e$ in panel a. (**c**) Amplitudes of the current steps $i_{s,i}$ at voltage $V_i = i \times hf/2e$ normalized by the critical current, I_c, as function of microwave power P. The traces are offset by $i \times 0.25 I_c$ for clarity.

S1.2. Shapiro step pattern

We provide additional plots of the Shapiro step data taken at $B = 0$ and $V_g = 0.5\,\mathrm{V}$ in Fig. S4. The data are taken for drive frequency $f = 1.6\,\mathrm{GHz}$ [c.f., the histogram in Fig. 1f of the main text]. The color-density plots in Fig. S4a&b depict maps of the differential resistance $\mathrm{d}V/\mathrm{d}I$ obtained by numerical differentiation of the $I - V$ characteristics (sweep direction positive to negative bias) as a function of microwave power P and voltage V (for S4a) or current I (for S4b), respectively. The resolution is limited by the number of microwave power values, we probed.

In Fig. S4c, we plot the amplitudes of the current steps $i_{s,i}$ at voltage $V_i = i \times hf/2e$, normalized by the critical current, I_c, as function of microwave power P. The suppression of the first and third steps, compared to the second and fourth steps, is clearly visible.

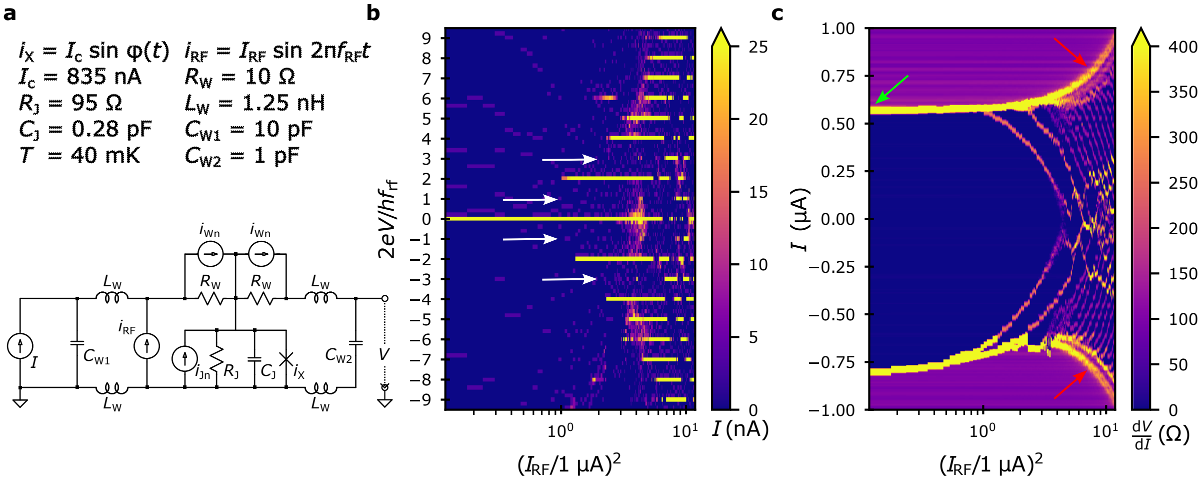

FIG. S5. **Shapiro simulation -** (**a**) Schematic of the simulated circuit and circuit element parameters. The current source i_{RF} imparts a microwave drive current with amplitude I_{RF} and frequency $f_{\mathrm{RF}} = 1.6\,\mathrm{GHz}$. (**b**) 2D map of the voltage histogram of Shapiro steps (bin size: $0.66\,\mu\mathrm{V}$) as a function of the square of the microwave drive amplitude I_{RF}^2 and DC averaged voltage V; first and third Shapiro steps are missing (positions indicated by arrows). (**c**) 2D map of the differential resistance $\mathrm{d}V/\mathrm{d}I$ obtained by numerical differentiation and plotted as a function of the square of the microwave drive amplitude I_{RF}^2 and the bias current I.

This indicates that the junction phase evolution cannot lock to the RF drive signal for Shapiro steps that correspond to odd-numbered multiples of the drive frequency. This effect was described theoretically for Josephson junctions with a 4π-periodic component in the current-phase relation, e.g., due to Landau-Zener transitions between low-energy Andreev bound states [8] or 4π-periodic mid-gap Andreev bound states. Details on the phase dynamics were given, e.g., in Refs. [9, 10]. The picture was extended to include the intrinsic dynamics of the hybridized Majorana bound states in Ref. [11].

An alternative mechanism for the suppression of Shapiro steps may occur in inductively-shunted Josephson junctions, as mentioned by, e.g., Sullivan, *et al.*, [12]. Our device is not shunted by a single lump-circuit element inductance but a series combination of wirebond inductances and the stray capacitances of a flatband connector that connects the sample mount with a copper-powder filter. The resulting environmental modes have characteristic frequencies in the low-GHz range. After becoming aware of the issue, we

have traced the presence of these modes in many previous experiments by us. In this frequency range, the shunt reactance becomes inductive, and period-doubling dynamics sets in. At even higher frequencies, the large geometric capacitance of shunts the junction and regular single-period dynamics is recovered. Thus all Shapiro steps are present at higher drive frequencies, as observed in experiments [13, 14].

To demonstrate that the suppression of Shapiro steps indeed occurs by this mechanism, we simulate a simplified version of the measurement circuit used in the experiments of Fig. S4. The RF shunt impedance of the environment is modeled by the inductances L_{W} (originating mostly from the wirebond connections to the chip-carrier) and the parasitic capacitances C_{W1} and C_{W2} (in the chip-carrier and flatband connector) [Fig. S5a]. The current and voltage connections are routed differently, thus we expect an imbalance between the lead pairs. We have estimated the magnitudes of capacitance and inductance values. For simplicity, we choose the inductances identical and adjust L_{W} such that the value of the $L_{\mathrm{W}}C_{\mathrm{J}}$-product fits with the in Section S1.1 inferred frequency of the circuit resonance. The imbalance in the leads is modeled by setting $C_{\mathrm{W1}} = 10\ C_{\mathrm{W2}}$, and the microwave drive current with amplitude I_{RF} and frequency $f_{\mathrm{RF}} = 1.6\,\mathrm{GHz}$ is sourced by the AC current source i_{RF}. The current sources $i_{(\circ)\mathrm{n}}$ add white noise with temperature T for each resistor in the circuit.

The results of the simulation are plotted in Fig. S5b&c. The first and third Shapiro steps are suppressed, indicated by the white arrows in the current histogram of Fig. S5b. The differential resistance map [Fig. S5c] shares characteristic features with the experimental data [Fig. S4b]: The $I-V$s are hysteretic. At low microwave amplitude, retrapping occurs at $\approx 540\,\mathrm{nA}$ [green arrow]. At high microwave amplitudes, a fringe feature is observed [red arrows]. By comparison with Fig. S4b&c, it can be traced back to the retrapping voltage $V_{\mathrm{r}} \approx 35\,\mu\mathrm{V} \approx hf_{\mathrm{LC}}/e$, where $f_{\mathrm{LC}} = 1/2\pi\sqrt{L_{\mathrm{W}}C_{\mathrm{J}}} = 8.5\,\mathrm{GHz}$ and is thus related to the circuit resonance.

S2. I-V CHARACTERISTICS OF THE RCL-SHUNTED JUNCTION FOR DIFFERENT RATIOS OF L_S/C_J

In measurement configuration C1, the LC-resonance produces several peaks in the $I-V$ characteristics of the externally shunted device. Whereas the value of the product $L_S \times C_J$ is calculated from the resonance frequency f_{LC}, the ratio L_S/C_J remains undetermined. We infer it by comparing the shape of simulated $I-V$ traces (i.e., the relative peak heights) to the experimental data. Fig. S6 depicts the measured trace and a series of simulated data for comparison. The best agreement is found for $L_S = 3.3\,\text{nH}$ and $C_J = 0.28\,\text{pF}$. These values match well with estimates based on the geometrical inductance of the wirebonds and the capacitance required to observe the $I-V$ hysteresis, cf. Section S1.1.

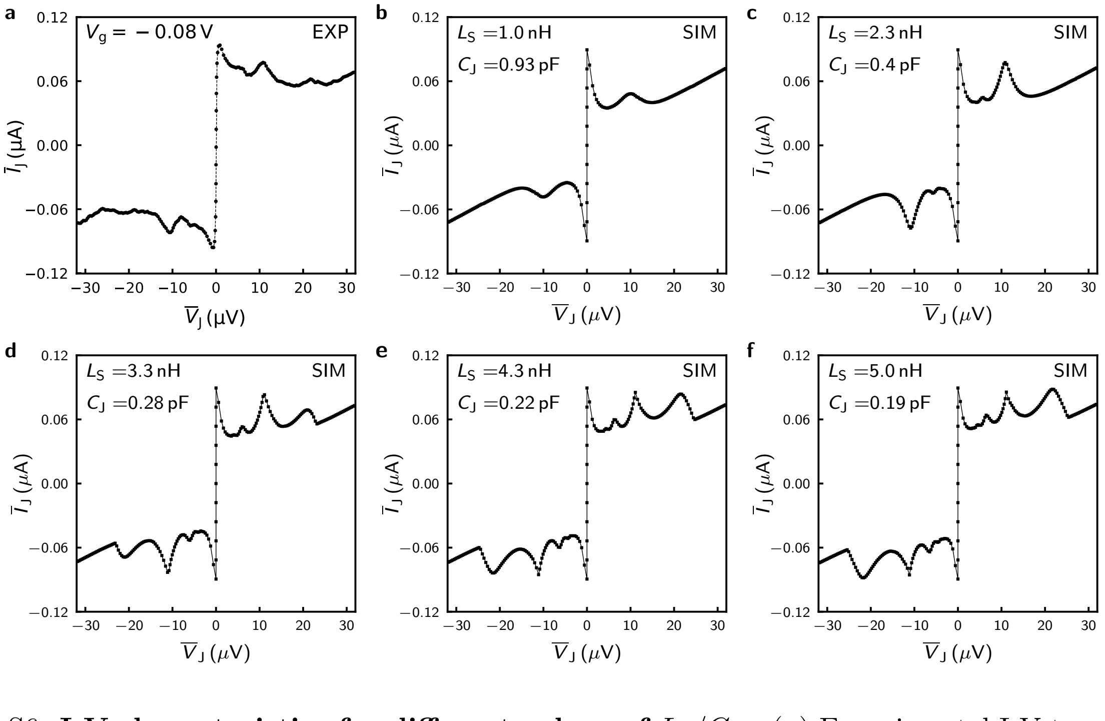

FIG. S6. **I-V characteristics for different values of** L_S/C_J **-** (**a**) Experimental I-V trace at $V_g = -0.08\,\text{V}$ and (**b**)-(**f**) simulated $\overline{I}_J(\overline{V}_J)$ for different values of L_S and C_J in the equivalent circuit of Fig. 2c of the main text. The ratio of L_S/C_J is varied while keeping the product $L_S \times C_J$ constant.

S3. BACKGROUND SUBTRACTION AND DATA NORMALIZATION

The gain of the amplification chain has a strong dependency on frequency due to model-dependent bandwidth limitations of the amplifiers and standing wave conditions in the wiring. This can be seen in Fig. S7 where we plot the spectral density of the unbiased Josephson junction, $\mathrm{PSD_d}(f_\mathrm{d}, \overline{V_\mathrm{J}} = 0)$, at $V_\mathrm{g} = 0.5\,\mathrm{V}$ in measurement configuration C1. Here, the largest contribution to the signal originates from the noise background of the low-temperature amplifier with effective noise temperature $\sim 2.3\,\mathrm{K}$. The gain oscillations are fully reproducible when repeating the measurement [Fig. S7b].

In order to resolve the amplitudes of emission lines, we subtract the noise background $\mathrm{PSD_d}(f_\mathrm{d}, \overline{V_\mathrm{J}} = 0)$ from the measured signal. This removes the effective noise of the amplifier and the thermal noise floor of the sample circuit.

To visualize the relative amplitudes of f_J and $f_\mathrm{J}/2$ emission signals in color-density

plots [e.g., Fig. 3a&e], we further divide the traces at fixed detector frequency f_d by the maximum value of the individual data trace; i.e., the normalized power spectral density

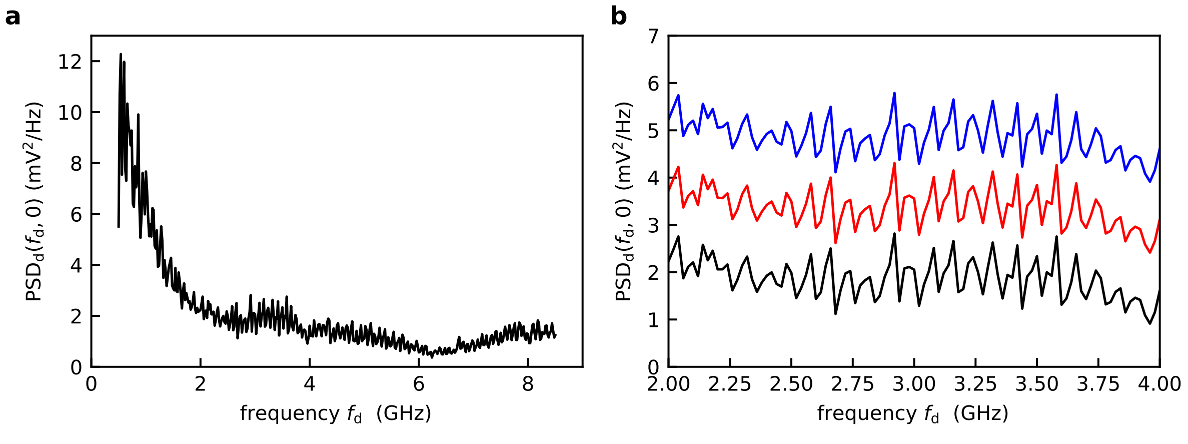

FIG. S7. **Background power spectral density -** (**a**) Power spectral density (PSD) of the unbiased Josephson junction at $V_\mathrm{g} = 0.5\,\mathrm{V}$ in circuit configuration C1 as measured by the spectrum analyzer (raw data). The detection bandwidth is set to 2 MHz. (**b**) Three individual measurements of the background PSD in the frequency range 2 GHz to 4 GHz plotted with offsets of $1.5\,\mathrm{mV^2/Hz}$. The gain oscillations (of the noise background) in $\mathrm{PSD_d}$ are fully reproducible.

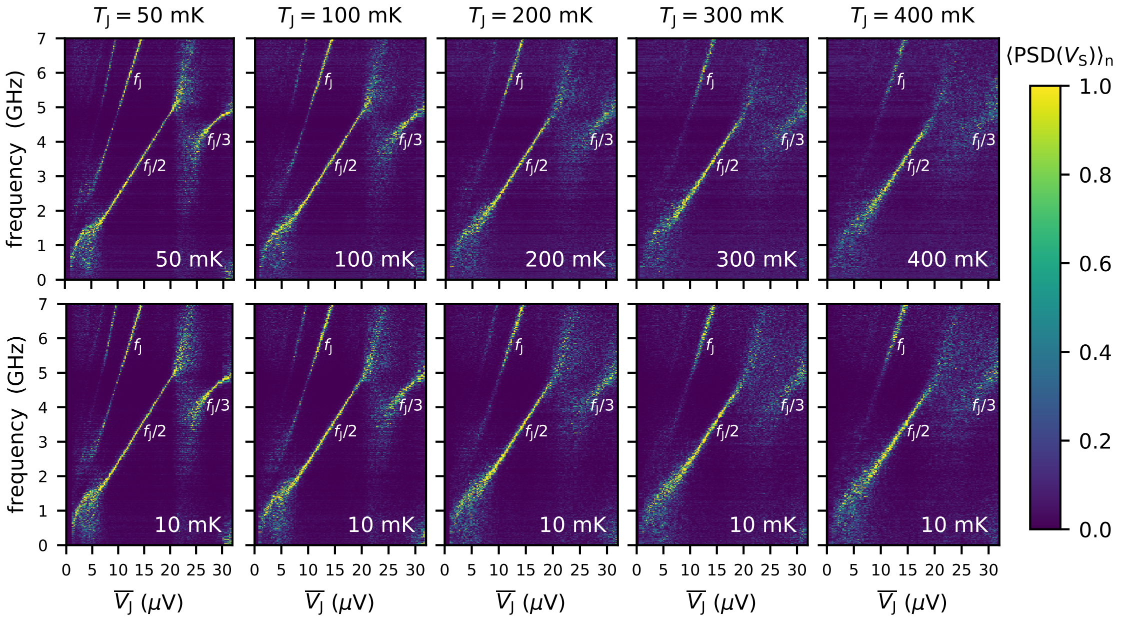

FIG. S8. **Josephson emission for different amounts of white noise -** Normalized power spectral density of $V_S(t)$, obtained by RCLSJ model simulations, as a function of $\overline{V_J}$. The circuit parameters are identical to Fig. 3e&g of the main text. The column title marks the noise temperature T_J, pertaining to resistor R_J, and the label in the panel indicates the noise temperature T_{sh} of R_S and R_W.

is defined as

$$\mathrm{PSD_d(normalized)} \equiv \frac{\mathrm{PSD_d}(f_d, \overline{V_J}) - \mathrm{PSD_d}(f_d, 0)}{\max\left(\mathrm{PSD_d}(f_d, \overline{V_J}) - \mathrm{PSD_d}(f_d, 0)\right)} . \tag{S2}$$

S4. ADDITIONAL NUMERICAL SIMULATIONS

In this section, we present additional numerical simulations, exploring the effects of white noise fluctuations, current-phase relation of the Josephson junction and loading by an external circuit.

S4.1. White noise fluctuations and linewidth of the Josephson emission

In Figs. 3c&g, only a small amount of white noise fluctuations has been added to broaden the emission lines for better visibility. Here, we further explore the effect of white noise on the width of the emission features and broadening of the circuit resonance features. The results of RCLSJ model simulations for different combinations of noise temperatures T_J [of the resistor R_J] and T_sh [of shunting resistors R_W and R_S] are plotted in Fig. S8. The remaining circuit parameters are chosen identical to Fig. 3e&g of the main text. We observe that the noise temperature T_J broadens the region around $\overline{V_\mathrm{J}} = hf_\mathrm{LC}/e$, for which the dynamics are strongly affected by the circuit resonance, whereas the width of the emission lines for intermediate bias scales approximately linearly with the temperature. We plot the linewidth of the $f_\mathrm{J}/2$ emission line for $\overline{V_\mathrm{J}} = 10\,\mu\mathrm{V}$ in Fig. S9. The linewidth is obtained by fitting a Lorentzian lineshape,

$$L(f, f_0) = p_0 + p_1 \frac{\Gamma^2}{(f - f_0)^2 + \Gamma^2}$$

where f_0 is the center frequency, Γ the linewidth, p_0 the background, and p_1 the magnitude. The linewidths increase with the noise temperature. However, there is a substantial discrepancy in linewidth between the RCLSJ simulations of the simplified circuit and the experiment.

Several aspects may influence the emission linewidth that are omitted in the simulations: Firstly, the subgap current is modeled by the lump-circuit resistor R_J, and white noise is assumed for the thermal noise of R_J. At finite voltage, HgTe Josephson junctions exhibit excess current transport by the multiple Andreev reflection mechanism [15–17]. In this process, multiple charge quanta are transported. Thus the shot noise contribution to white noise is enhanced. The current fluctuations are given by $\langle i^2 \rangle = 2q_\mathrm{eff} I_\mathrm{qp}$, where $q_\mathrm{eff} = q_\mathrm{eff}(V_\mathrm{J})$ is the voltage dependent effective charge, and I_qp is quasiparticle component of the junction current. The charge q_eff can be $\gg e$, as has been demonstrated for pin-holes in tunnel junctions [18] or diffusive SNS devices [19, 20]. We thus may expect a

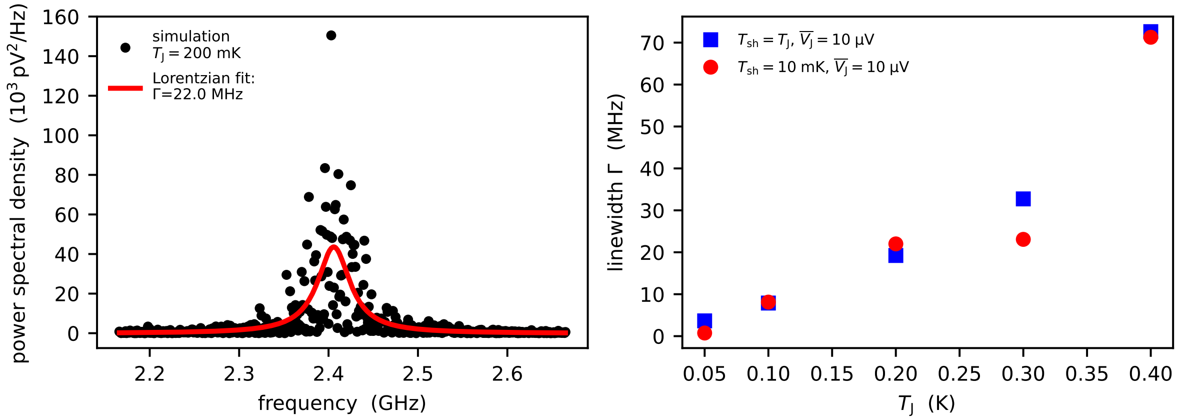

FIG. S9. **Linewidth of the Josephson radiation at** $f_J/2$ **-** (**a**) Power spectral density of $V_S(t)$, obtained by RCLSJ model simulations for parameters $\overline{V_J} = 10\,\mu V$, $T_J = 200\,mK$ and $T_{sh} = 10\,mK$, plotted against frequencies in a narrow range around f_J. The circuit parameters are identical to Fig. 3e&g of the main text. The solid red line is a Lorentzian fit to the data. (**b**) Linewidths of the Josephson emission at frequency f_J for $\overline{V_J} = 10\,\mu V$ extracted from the numerical simulations in Fig. S8 (second row) by fitting a Lorentzian curve to the data (solid squares).

significant contribution to white noise in our experiment. However, for a proper quantification of the shot noise contribution, separate noise measurements need to be conducted. We have not measured the shot noise in this device.

Secondly, emission lines are broadened when photons are exchanged with the environment; e.g., see Ref. [21]. A basic assessment of coupling to the detector circuit is presented in Sec. S4.3. A more comprehensive modeling including cavity modes in the RF fixture and circuit resonances in the external RF circuit could prove useful.

Lastly, real circuit elements and EM environments have intrinsic microwave loss. We have conducted first tests on co-planar microwave resonator structures on CdTe substrates. The preliminary results indicate substantial, material-specific microwave loss in structures fabricated on CdTe. A systematic study is currently underway.

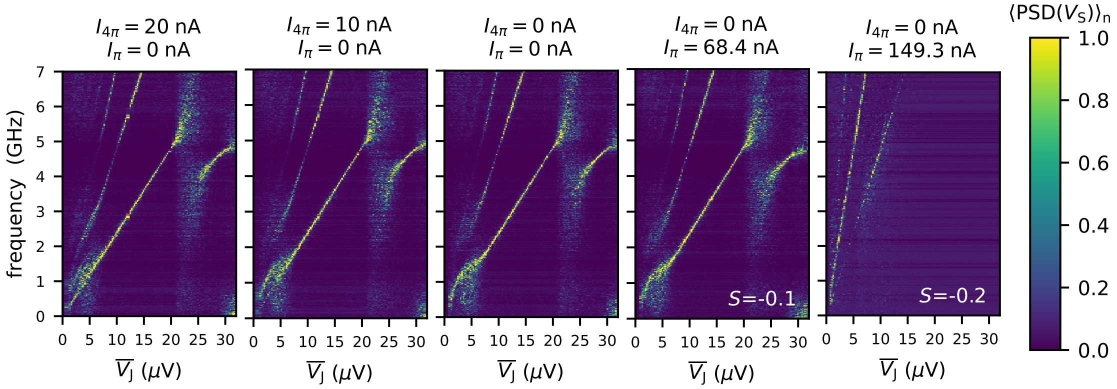

FIG. S10. **Josephson radiation and current-phase relation -** Normalized power spectral density of $V_S(t)$, obtained by RCLSJ model simulations, as a function of $\overline{V_J}$. The critical current is $I_c = 835\,\mathrm{nA}$, and $T_J = T = 50\,\mathrm{mK}$. The remaining circuit parameters are identical to Fig. 3e&g of the main text. The column title indicates the supercurrent components in Eq. S4 S4.2, and the label in the panel displays the skewness of the CPR.

S4.2. Current-phase relation

In this section, we explore the influence of the current-phase relation (CPR) on the Josephson emission in the limit when period-doubling dynamics is relevant. In Fig. S10, we plot a series of PSD maps of $V_S(T)$ for RCLSJ models with different CPRs. Here, the CPRs are parametrized by

$$I_X(\varphi) = I_{4\pi} \sin \varphi/2 + I_{2\pi} \sin \varphi + I_{\pi} \sin 2\varphi \ . \tag{S3}$$

The remaining simulation parameters correspond to the device at $V_g = 0.5\,\mathrm{V}$ in circuit configuration C1 [cf. Fig. 3e&g of the main text]. The critical current $I_c = \max_\varphi I_x X(\varphi)$ is kept equal to 835 nA. In the first two panels on the L.H.S., a small 4π-periodic supercurrent is added. The magnitude of this supercurrent component is chosen to reflect the estimated supercurrent carried by QSH edge channels [14]. The two panels on the R.H.S., feature CPRs with a $\sin 2\varphi$ component and the skewness, $S = (2\varphi_{\max} - \pi)/\pi$, indicated by the label in the respective panels. We observe that a small addition of π-

or 4π-periodic supercurrent does not lead to qualitative changes in the expected emission patterns. Conversely, the simulation with the larger $\sin 2\varphi$ component and $S = -0.2$ displays a distinctly different emission pattern. Here, the emission has a strong $2f_\mathrm{J}$ component. The features pertaining to the LC-resonance and emission at fractions of the Josephson frequency are absent.

S4.3. Coupling via the bias tee

Lastly, we study the effect of coupling the detector circuit to the sample. The connection is made by a wirebond with inductance L to a PCB board and further via a coax cable and bias tee to a low-temperature pre-amplifier with an input impedance of $50\,\Omega$. The bias tee contains a coupling capacitor C. We simulated the detector circuit by loading the sample circuit at node V_S with an LCR series circuit and analyze the voltage drop V_S^* across the resistor $R = 50\,\Omega$. We vary the values of L and C to account for different bias tee and wirebond inductance combinations. The remaining simulation parameters

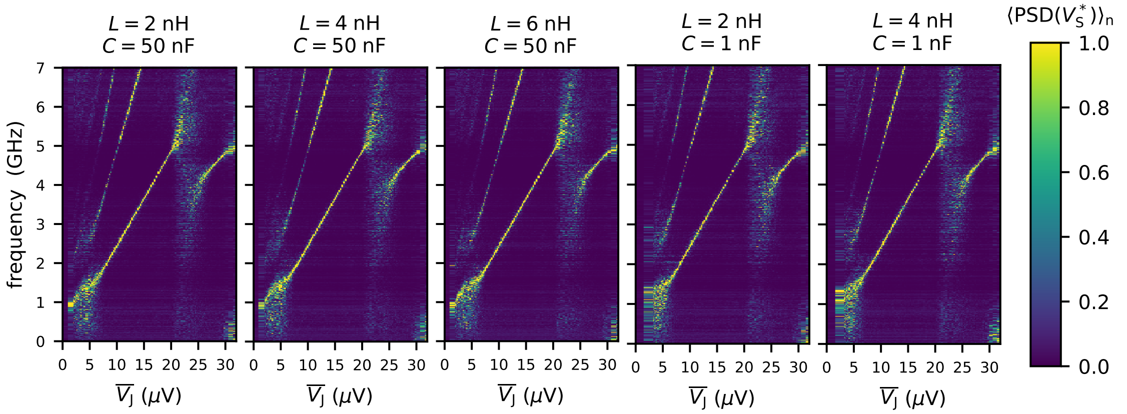

FIG. S11. **Circuit loading by the detection circuit -** Normalized power spectral density of $V_\mathrm{S}(t)$, obtained by RCLSJ model simulations, as a function of $\overline{V_\mathrm{J}}$. The circuit parameters are identical to Fig. 3e&g of the main text, and the noise temperatures are set to $T_\mathrm{J} = T = 50\,\mathrm{mK}$. The detector is coupled to the sample circuit via a series LC circuit. The column title indicates parameters of the LC circuit.

correspond to the device at $V_g = 0.5\,\mathrm{V}$ in circuit configuration C1 [cf. Fig. 3e&g of the main text]. We find no qualitative difference in the emission patterns.

S5. ADDITIONAL MEASUREMENTS ON DEVICE QC675JJ1

Data on a second device in side-contacted geometry are presented in Fig. S12. The device has no gate thus only a limited number of measurements have been performed. In this experiment, the linewidth of the emission is smaller, and resonance features are more pronounced. Both properties are indicative of a higher Q-factor of the RCL-circuit. At frequencies above $\approx 4\,\mathrm{GHz}$, additional features become visible. The voltage values and frequencies are consistent with a down-conversion by a circuit resonance; i.e., the features are found at $f_J(\overline{V_J}) - f_{res}$ and $f_J(\overline{V_J})/2 - f_{res}$, where $f_{res} \approx 4\,\mathrm{GHz}$. The presence of an environmental resonance also reveals itself as a peak in the $I - V$ characteristic [Fig. S12b] at $\overline{V_J} = hf_{res}/e \approx 16.5\,\mathrm{\mu V}$. Whereas the device in the main text does not exhibit $f_J/3$ emission clearly, it can be observed in the dataset of Fig. S12a. The mech-

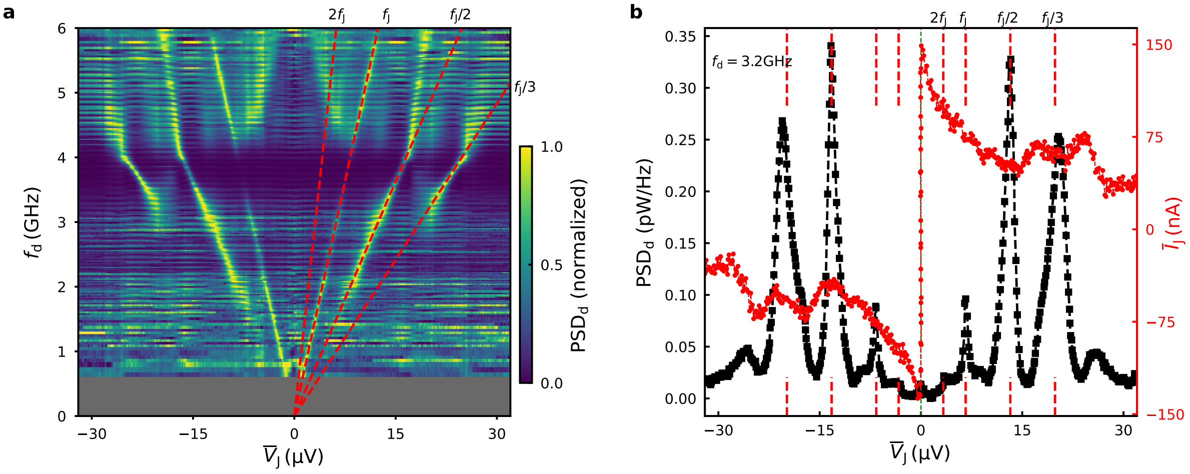

FIG. S12. **Josephson emission (device QC675JJ1) -** (**a**) Normalized PSD as a function of junction voltage, $\overline{V_J}$. Emission features are observed at f_J, $f_J/2$, and $f_J/3$. (**b**) Power spectral density $\mathrm{PSD_d}$ recorded at detector frequency $f_d = 3.2\,\mathrm{GHz}$ and junction current $\overline{I_J}$ as a function of the voltage drop across the junction, $\overline{V_J}$. The parameters of the DC circuit analysis are $I_c = 148\,\mathrm{nA}$, $R_S = 8.23\,\Omega$, and $R_W = 4.74\,\Omega$.

anism behind $f_J/3$-emission is identical to period-doubling dynamics. Phase evolution with 6π-periodicity is enabled at high bias in inductively-shunted device for large enough β_c [22].

---

[1] R. L. Kautz and J. M. Martinis, Noise-affected I-V curves in small hysteretic Josephson junctions, Physical Review B **42**, 9903 (1990).

[2] W. C. Stewart, Current-Voltage Characteristics of Josephson Junctions, Applied Physics Letters **12**, 5 (1968).

[3] D. E. McCumber, Effect of ac Impedance on dc Voltage-Current Characteristics of Superconductor Weak-Link Junctions, Journal of Applied Physics **39**, 3113 (1968).

[4] Y. C. Chen, M. P. A. Fisher, and A. J. Leggett, The return of a hysteretic Josephson junction to the zero-voltage state: I-V characteristic and quantum retrapping, Journal of Applied Physics **64**, 3119 (1988).

[5] H. Courtois, M. Meschke, J. T. Peltonen, and J. P. Pekola, Origin of Hysteresis in a Proximity Josephson Junction, Physical Review Letters **101**, 067002 (2008).

[6] K. Le Calvez, L. Veyrat, F. Gay, P. Plaindoux, C. B. Winkelmann, H. Courtois, and B. Sacépé, Joule overheating poisons the fractional ac Josephson effect in topological Josephson junctions, Communications Physics **2**, 1 (2019).

[7] D. S. Antonenko and M. A. Skvortsov, Quantum decay of the supercurrent and intrinsic capacitance of Josephson junctions beyond the tunnel limit, Physical Review B **92**, 214513 (2015).

[8] M. C. Dartiailh, J. J. Cuozzo, B. H. Elfeky, W. Mayer, J. Yuan, K. S. Wickramasinghe, E. Rossi, and J. Shabani, Missing Shapiro steps in topologically trivial Josephson junction on InAs quantum well, Nature Communications **12**, 78 (2021).

[9] F. Domínguez, O. Kashuba, E. Bocquillon, J. Wiedenmann, R. S. Deacon, T. M. Klapwijk, G. Platero, L. W. Molenkamp, B. Trauzettel, and E. M. Hankiewicz, Josephson junction dynamics in the presence of 2π- and 4π-periodic supercurrents, Physical Review B **95**,

195430 (2017).

[10] J. Park, Y.-B. Choi, G.-H. Lee, and H.-J. Lee, Characterization of Shapiro steps in the presence of a 4π-periodic Josephson current, Phys. Rev. B **103**, 235428 (2021).

[11] Z. Wang, J.-J. Feng, Z. Huang, and Q. Niu, Transport Theory for Topological Josephson Junctions with a Majorana Qubit, Phys. Rev. Lett. **129**, 257001 (2022).

[12] D. B. Sullivan, R. L. Peterson, V. E. Kose, and J. E. Zimmerman, Generation of Harmonics and Subharmonics of the Josephson Oscillation, Journal of Applied Physics **41**, 4865 (1970).

[13] J. Wiedenmann, E. Bocquillon, R. S. Deacon, S. Hartinger, O. Herrmann, T. M. Klapwijk, L. Maier, C. Ames, C. Brüne, C. Gould, A. Oiwa, K. Ishibashi, S. Tarucha, H. Buhmann, and L. W. Molenkamp, 4π-periodic Josephson supercurrent in HgTe-based topological Josephson junctions, Nat Commun **7**, 10303 (2016).

[14] E. Bocquillon, R. S. Deacon, J. Wiedenmann, P. Leubner, T. M. Klapwijk, C. Brüne, K. Ishibashi, H. Buhmann, and L. W. Molenkamp, Gapless Andreev bound states in the quantum spin Hall insulator HgTe, Nat Nano **12**, 137 (2017).

[15] M. Octavio, M. Tinkham, G. E. Blonder, and T. M. Klapwijk, Subharmonic energy-gap structure in superconducting constrictions, Physical Review B **27**, 6739 (1983).

[16] K. Flensberg, J. B. Hansen, and M. Octavio, Subharmonic energy-gap structure in superconducting weak links, Physical Review B **38**, 8707 (1988).

[17] G. Niebler, G. Cuniberti, and T. Novotnỳ, Analytical calculation of the excess current in the Octavio–Tinkham–Blonder–Klapwijk theory, Superconductor Science and Technology **22**, 085016 (2009).

[18] P. Dieleman, H. G. Bukkems, T. M. Klapwijk, M. Schicke, and K. H. Gundlach, Observation of Andreev Reflection Enhanced Shot Noise, Physical Review Letters **79**, 3486 (1997).

[19] P. Roche, H. Perrin, D. C. Glattli, H. Takayanagi, and T. Akazaki, Enhanced shot noise in long quasi-diffusive S–N–S junctions, Physica C: Superconductivity **352**, 73 (2001).

[20] F. Lefloch, C. Hoffmann, D. Quirion, and M. Sanquer, Shot noise in diffusive SNS and SIN junctions, Physica E: Low-dimensional Systems and Nanostructures 23rd International Conference on Low Temperature Physics (LT23), **18**, 17 (2003).

[21] M. Hofheinz, F. Portier, Q. Baudouin, P. Joyez, D. Vion, P. Bertet, P. Roche, and D. Esteve, Bright Side of the Coulomb Blockade, Phys. Rev. Lett. **106**, 217005 (2011).

[22] E. Neumann and A. Pikovsky, Slow-fast dynamics in Josephson junctions, The European Physical Journal B - Condensed Matter **34**, 293 (2003).
